# All-inside versus complete tibial tunnel techniques in anterior cruciate ligament reconstruction: a systematic review and meta-analysis of randomized controlled trials

**DOI:** 10.1186/s13018-023-03613-y

**Published:** 2023-02-21

**Authors:** XiaoYu Lv, Ming Wang, TianYu Zhao, Ling Wang, ShuangBin Dong, HongBo Tan

**Affiliations:** 1 Kunming Medical University, Kunming, 650032 Yunnan People’s Republic of China; 2 Department of Orthopedic Surgery, 920th Hospital of Joint Logistics Support Force, 212 Daguan Road, Kunming, 650100 Yunnan People’s Republic of China

**Keywords:** Anterior cruciate ligament, Reconstruction, All-inside, Complete tibial tunnel, Tibial tunnel

## Abstract

**Background:**

All-inside anterior cruciate ligament reconstruction (ACLR) is a novel technique that has gained attention due to its minimally invasive. However, evidence surrounding the efficacy and safety between all-inside and complete tibial tunnel ACLR are lacking. Present work was aimed to compare clinical outcome for ACLR performed with an all-inside versus a complete tibial tunnel technique.

**Methods:**

Systematic searches were conducted of published literature on PubMed, Embase, and Cochrane for studies according to Preferred Reporting Items for Systematic Reviews and Meta-Analyses guidelines up to May 10, 2022. The outcomes included KT-1000 arthrometer ligament laxity test, International Knee Documentation Committee (IKDC) subjective score, Lysholm score, Tegner activity scale, and Knee Society Score (KSS) Scale, and tibial tunnel widening. Complications of interest extracted were graft re-ruptures and evaluated the graft re-rupture rate. Data from published RCTs meeting inclusion criteria were extracted and analyzed, and all the extracted data are pooled and analyzed by RevMan 5.3.

**Results:**

A total of 8 randomized controlled trials involving 544 patients (consisting of 272 all-inside and 272 complete tibial tunnel patients) were included in the meta-analysis. We found clinical outcomes (International Knee Documentation Committee [IKDC] subjective score: mean difference [MD], 2.22; 95% CI, 0.23–4.22; *p* = 0.03; Lysholm score: MD, 1.09; 95% CI, 0.25–1.93; *p* = 0.01; Tegner activity scale: MD, 0.41; 95% CI, 0.11–0.71; *p* < 0.01; Tibial Tunnel Widening: MD = − 1.92; 95% CI, − 3.58 to − 0.25; *p* = 0.02; knee laxity: MD = 0.66; 95% CI, 0.12–1.20; *p* = 0.02; and graft re-rupture rate: RR, 1.97;95% CI, 0.50–7.74; *P* = 0.33) in the all-inside and complete tibial tunnel group. The findings also indicated that all-inside may be more advantageous in tibial tunnel healing.

**Conclusion:**

Our meta-analysis indicated that the all-inside ACLR was superior to complete tibial tunnel ACLR in functional outcomes and tibial tunnel widening. However, the all-inside ACLR was not entirely superior to complete tibial tunnel ACLR in knee laxity measured, and graft re-rupture rate.

## Introduction

The anterior cruciate ligament (ACL) is one of the main stable structures of the knee joint, and mainly the effect is to limit tibial anteriorness [1]. With an annual incidence of 1 in 3000, this results in over 175,000 ACL injuries each year in the USA [2]. ACL rupture mostly occurs in young athletes. The ACL rupture is the main cause of the end of athletes' careers [3, 4], and often lead to knee instability, and easy to cause meniscus and cartilage damage [5, 6]. Arthroscopic reconstruction of the ACL is the most important method for ACL rupture [7]. The goals of ACL reconstruction are to restore knee stability and reduce post-traumatic meniscal tears and cartilage degradation [6].

The all-inside technique of ACLR is defined as creating the bone socket from the articular side of the tibia rather than conventional complete tibial tunnel through the knee joint and outer cortex [8]. The all-inside technique for ACLR, compared to the traditional Antero-Medial (AM) or Transtibial methods, features substantial improvements including two closed-socket tunnels, tibial suspensory fixation and smaller skin incisions [9]. In addition, all-inside ACLR technique with single semitendinosus tendon can basically meet the requirements of graft diameter and length, while the complete tibial tunnel technique requires double gracilis and semitendinosus [10].The all-inside proposed benefits include reduced incidence of complications such as tibial tunnel fractures; anatomic placement of the tibial tunnel; increased postoperative muscle, tendon, and bone preservation; and improved in long-term function [11–14]. The all-inside technique may be a valuable option for younger patients with open growth plate in order to preserve and guarantee a physiological skeletal growth [15]. Goyal et al. [16] had described the all-inside ACLR has the advantages, lesser early postoperative pain with similar clinical and functional outcomes compared to the complete tibial tunnel. Darren et al. [10] showed that the all-inside ACLR shows potential as a minimally invasive approach given the low graft failure rates and short-term improvements in knee function and stability, pain and patient important outcomes from this approach.

However, whether the all-inside technique is superior to tradition complete tibial tunnel technique remains controversial. The systematic review and meta-analysis aimed to compare clinical outcomes between all-inside ACLR with complete tibial tunnel ACLR with regard to function, knee stability, graft failure, and tibial tunnel widening. We hypothesis that the all-inside technique was superior to the complete tibial tunnel technique in clinical outcomes.

## Methods

### Literature search

This meta-analysis was performed according to the PRISMA (Preferred Reporting Items for Systematic Reviews and Meta-Analyses) criteria [17].

A comprehensive search was conducted across multiple databases (Cochrane, EMBASE and PubMed) from the date of database inception to May 10, 2022. The Medical Subject Headings and Boolean operator terms used for the search were [(“Anterior cruciate ligament” OR “ACL”) AND (“All-inside” OR “Suspensory fixation”)]. Identified articles and their corresponding references were reviewed according to the selection criteria for consideration of inclusion.

### Eligibility criteria

All articles that had an RCT study design and compared the clinical outcomes of ACLR with all-inside ACLR and complete tibial tunnel ACLR were considered for inclusion. Non–English language studies, case reports, review articles, meta-analysis, animal researches, or unpublished studies, and studies not directly comparing the outcomes of all-inside ACLR and complete tibial tunnel ACLR were excluded. Two independent authors (XY.L and MW) reviewed records retrieved from the initial search twice and excluded irrelevant ones. Titles and abstracts of remaining articles were then screened against the inclusion criteria. Included articles were critically reviewed according to a predefined data extraction form. Differences in opinions were resolved by open discussion between the first 3 authors (TY.Z).

### Data collection

Extracted data parameters included details on study design, publication year, patient numbers, basic demographics, follow-up times, surgical techniques, fixation methods, functional outcomes, and complications. Functional outcomes extracted included KT-1000 arthrometer ligament laxity test, International Knee Documentation Committee (IKDC) score, Lysholm score, Tegner activity scale, and Knee Society Score (KSS) Scale, and tibial tunnel widening. Complications of interest extracted were graft re-ruptures and evaluated the graft re-rupture rate. Data extracted were copied and organized into a Microsoft Excel spreadsheet. Metrics evaluated encompassed IKDC, Lysholm, Tegner, and KSS Scale, and graft re-rupture.

### Methodology assessment

Methodological quality of included studies was assessed with the Cochrane risk-of-bias tool for RCTs by 2 independent reviewers [18]. We used 7 criteria to assess RCTs, and each criterion was scored in 3 categories—low risk, high risk, or unclear risk of bias—before the quality of the RCT was determined according to Agency for Healthcare Research and Quality (AHRQ) standards.

### Statistical analysis

The mean difference (MD) and risk ratio (RR), along with their accompanying 95% CIs, were used as summary statistics for continuous and noncontinuous variables, respectively. This meta-analysis tested both fixed-effects and random-effects models. The fixed-effects model assumed equivalent treatment effects in an individual study, whereas the random-effects model assumed the presence of variations between studies. Chi-square tests were used to study heterogeneity between trials. The I2 statistic primarily estimated the percentage of total variation across studies owing to heterogeneity rather than chance. When I2 < 50%, the heterogeneity was evaluated to be low, and a fixed-effects model was used for the meta-analysis. Otherwise, a random-effects model was used. The final results of this study were reported using an inverse variance statistical method. Review Manager (Version 5.3) was used for statistical analysis.

## Results

### Literature search

**Fig. 1:**
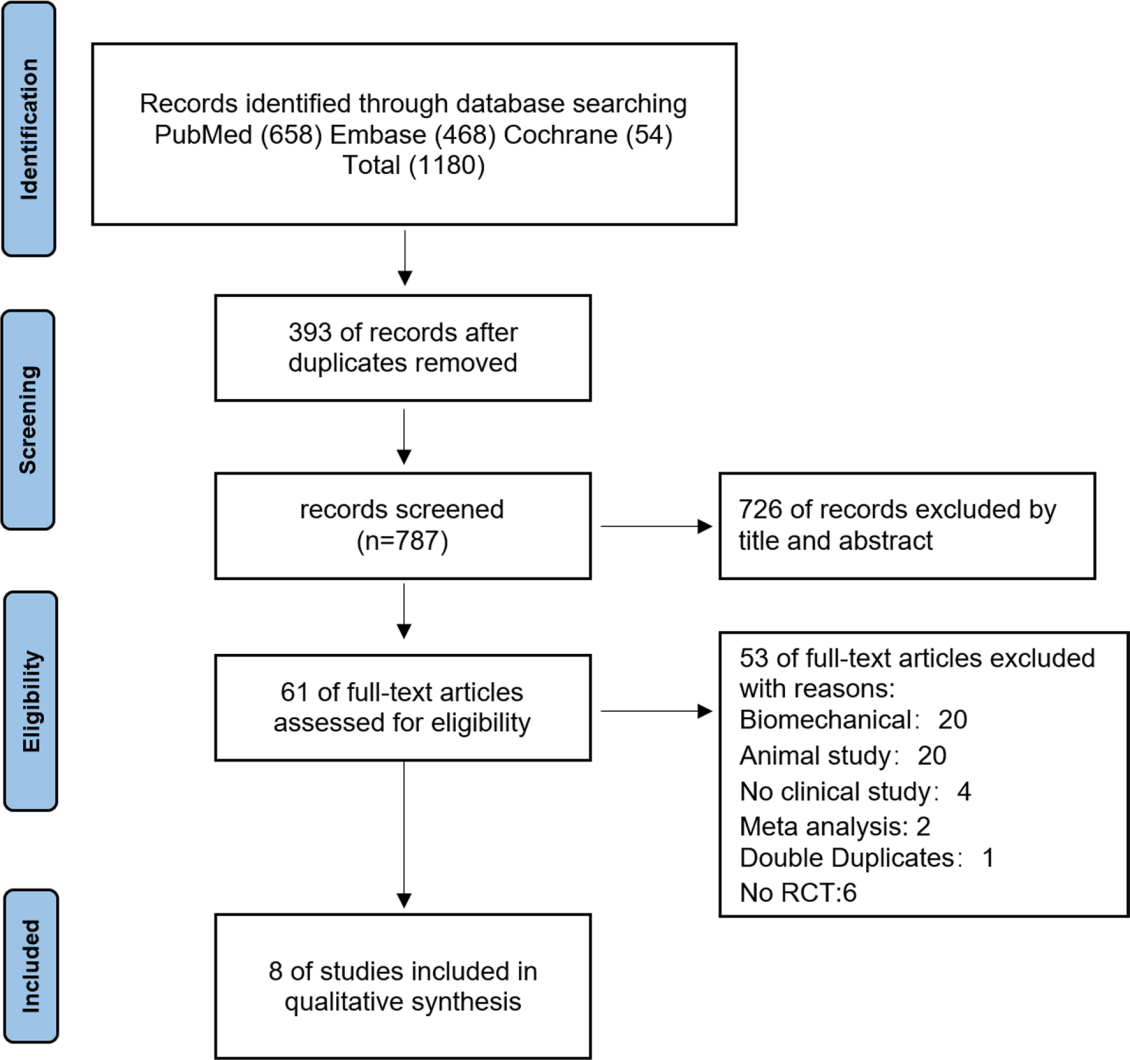
Preferred reporting items for systematic reviews and meta-analysis (PRISMA) flow diagram for the searching and identification of included studies

A PRISMA selection flowchart to identify included studies is illustrated in Fig. 1. A total of 1180 studies were identified from the initial search, of which 393 duplicates language articles were removed. Titles and abstracts of 787 remaining studies were screened in accordance with the predefined inclusion criteria, and 726 studies were excluded. Finally,61 full-text articles were assessed for eligibility. The following articles were excluded for the following reasons: Biomechanical study (20); Animal study (20); No clinical study (4); Meta analysis (2); Double Duplicates (1); No RCT study (6). Eventually, 8 RCTs were included [11, 19–25].

### Methodology assessment

**Fig. 2:**
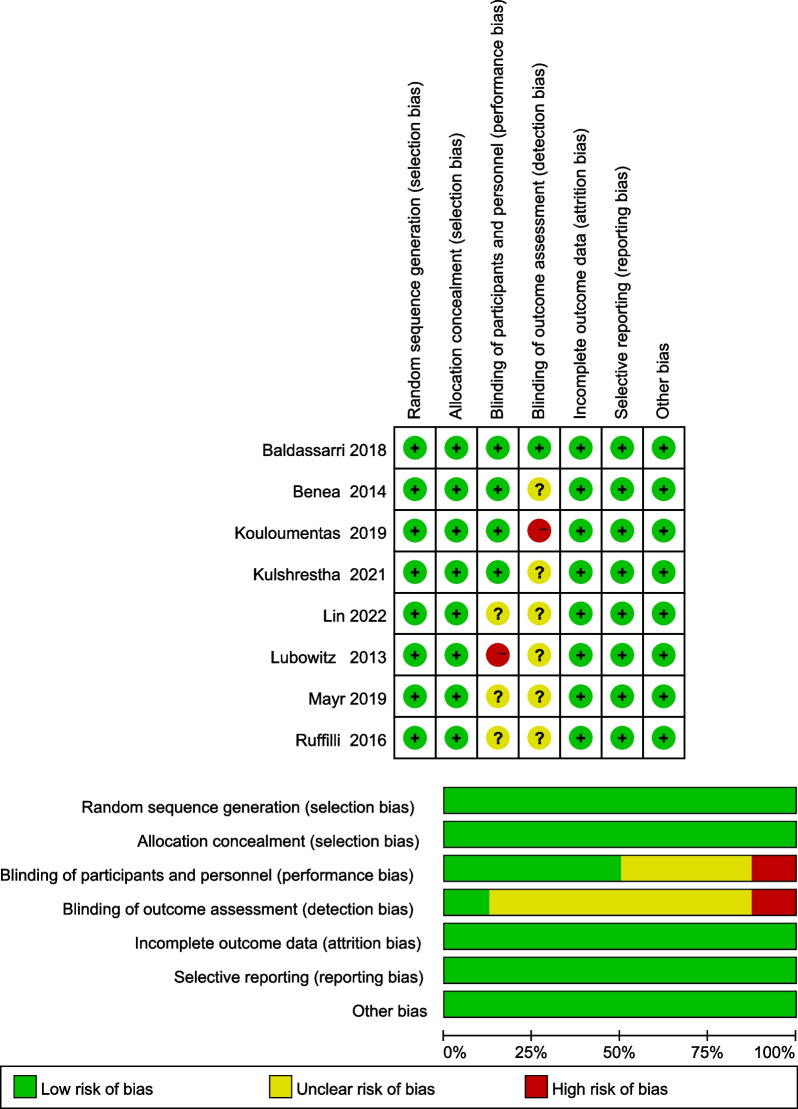
Summary of the risk-of-bias assessment for the included studies

In terms of design quality, the included studies were all RCTs. All included allocation concealment and blinding in the design, increasing the reliability of the results. A study [23] no blinding of participants and personnel, and a study [20] no blinding of outcome assessment. The Cochrane risk-of-bias assessment for all 7 RCTs is shown in Fig. 2. Overall, the quality of the design of the included studies was ideal.

### Demographic characteristics

A total of 272 All-inside patients and 272 complete tibial tunnel patients were included in this study. All except 1 study [11] had a 12 months minimum follow-up period, with mean follow-up ranged from 6 [11] to 48 [19] months. The study characteristics are presented in Table (Table 1).

**Table 1:** Baseline demographic characteristics and details of individual randomized controlled trials

						All-inside			Complete tibial tunnel				
Study	Registration Number	Local	Period	Study design, Level of Evidence	SEX male/female n	No. of patients	Age, year	Graft type	No. of patients	Age, year	Graft type	Follow-up, mouth	Outcome measurement
Baldassarri 2019	NR	Italy	November 2012 and September 2013	RCT; Level 2	46/13	28	24.7	NR	31	25.2	NR	48	Marx, Tegner activity score, IKDC score, and return to sport
Benea 2014	NCT01448278	France	2010 to 2011	RCT; Level 1	29/17	23	28.4 ± 8.6	ST4	23	30.2 ± 9.4	DGST	6	IKDC, VAS score, knee laxity assessment (use KT-1000 arthrometer), tunnel position measured with X-ray
Kouloumentas 2019	NR	Greece	2015 to 2016	RCT; level 1	55/35	45	27.6 ± 11.4	ST4	45	29.7 ± 11.0	DGST	24	Lysholm, IKDC, KOOS, KSS score, knee laxity assessment (use KT-1000 arthrometer), isokinetic testing, and graft failure
Kulshrestha 2021	NR	India	January 01, 2016, to December 31, 2016	RCT,Level 1	NR	40	29.25 ± 5.47	ST4	40	33.00 ± 5.23	ST4	24	Tegner activity scale, VAS and European Quality of Life Index(EQ-5D), KSS Score
Lin 2022	ChiCTR1800018543	China	September 2018 to July 2019	RCT,Level 1	45/6	25	31.3 ± 5.8	ST4	26	29.9 ± 4.6	DGST	12	Lysholm, Tegner activity score, IKDC scores GNRB arthrometer to assess knee laxity, Graft maturity on MRI
Lubowitz 2013	NR	America	NR	RCT; Level 1	77/71	75	39.3 ± 12.1	NR	73	41.1 ± 10.8	NR	24	IKDC Knee Examination Form, IKDC Subjective Knee Evaluation Form, KSS for pain and function, SF-12 score, tibial tunnel measured using X-ray,acetaminophene oxycodone hydrochloride (or equivalent) intake, and VAS pain score
Mayr 2019	NCT01755819	Austria	2013 to 2016	RCT; Level 2	21/9	16	25 ± 6	ST4	14	29 ± 7	DGST	24	Pivot shift, Tegner activity score, Lysholm s, IKDC score, knee laxity assessment (use KT-1000 arthrometer), hop testing, Tunnel volume diameter and location measured with CT
Ruffilli 2016	NR	Italy	NR	RCT; NR	32/8	20	NR	HG	20	NR	HG	24	IKDC score,Tegner activity scale, Graft ligamentization score assessed with MRI

*NR* No report, *ST4* Quadrupled semitendinosus tendon, *DGST* Doubled gracilis and semitendinosus tendons, *HG* Hamstring graft, *IKDC* International Knee Documentation Committee, *KOSS* Knee injury and osteoarthritis score, *KSS* Knee society score, *CT* Computed tomography, *VAS* Visual analog score, *SF-12* Short form 12, *MRI* Magnetic resonance imaging

### Clinical outcomes and complications

#### IKDC subjective score

**Fig. 3:**
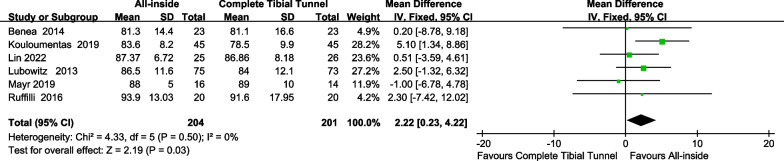
Forest plot for meta-analysis of IKDC subjective score between the all-inside and complete tibial tunnel groups

The final IKDC subjective scores were obtained from the last follow-up of each study. Six studies [11, 20, 22–25] reported IKDC subjective score, and no heterogeneity was found (*p* = 0.50; I2 = 0%). The fixation effect model was used, and the results showed that there was no significant difference between the 2 groups (MD = 2.22; 95% CI, 0.23–4.22; *p* = 0.03), indicating no difference in Final IKDC Subjective scale between the study and control groups (Fig. 3).

#### Lysholm scale

**Fig. 4:**
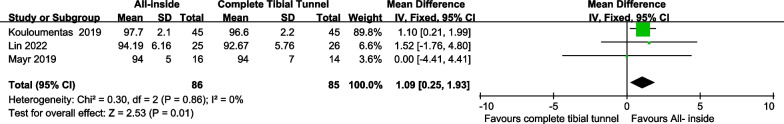
Forest plot for meta-analysis of Lysholm score between the all-inside and complete tibial tunnel groups

The Lysholm Scores were obtained from the last follow-up of each study. Three studies [20, 22, 24] reported Lysholm score, and no heterogeneity was found (*p* = 0.86; I2 = 0%). The fixation effect model was used, and the results showed that there was a significant difference between the 2 groups (MD = 1.09; 95% CI, 0.25–1.93; *p* = 0.01), indicating that the All-inside group was superior to the complete tibial tunnel group in Lysholm score (Fig. 4).

#### Tegner activity scale

**Fig. 5:**
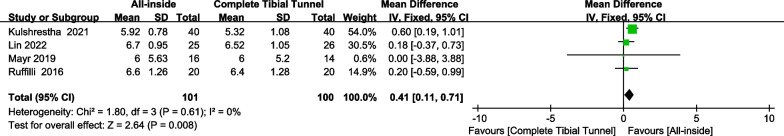
Forest plot for meta-analysis of Tegner score between the all-inside and complete tibial tunnel groups

The Tegner activity scale is used to evaluate activity and sporting levels [26]. It was used in conjunction with the Lysholm Knee Scoring scale to assess knee function. Four studies [21, 22, 24, 25] reported Tegner scale, and no heterogeneity was found (*p* = 0.61; I2 = 0%). The fixation effect model was used, and the results showed that there was a significant difference between the 2 groups (MD = 0.41; 95% CI, 0.11–0.71; *p* < 0.01), indicating that the All-inside group was superior to the complete tibial tunnel group in Tegner score (Fig. 5).

#### KSS scale

**Fig. 6:**
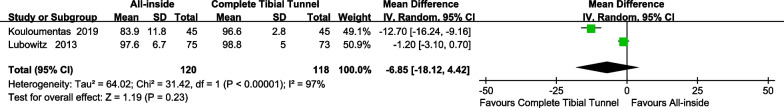
Forest plot for meta-analysis of KSS score between the all-inside and complete tibial tunnel groups

The Knee Society Score (KSS) Scale was obtained from the last follow-up of each study. Two studies [20, 23] reported KSS scale, and no heterogeneity was found (*p* < 0.01; I2 = 97%). The random effect model was used, and the results showed that there was no significant difference between the 2 groups (MD = − 6.85; 95% CI, − 18.12–4.42; *p* = 0.23), indicating no difference in KSS score between the study and control groups (Fig. 6).

#### Tibial tunnel widening (mm)

**Fig. 7:**
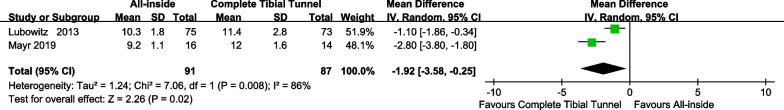
Forest plot for meta-analysis of tibial tunnel widening (mm) between the all-inside and complete tibial tunnel groups

Two studies [23, 24] reported tibial tunnel widening data, and no heterogeneity was found (*p* < 0.01; I2 = 86%). The random effect model was used, and the results showed that there was no significant difference between the 2 groups (MD = − 1.92; 95% CI, − 3.58 to − 0.25; *p* = 0.02), indicating no difference in tibial tunnel widening data between the study and control groups (Fig. 7).

#### Knee laxity measured (mm)

**Fig. 8:**
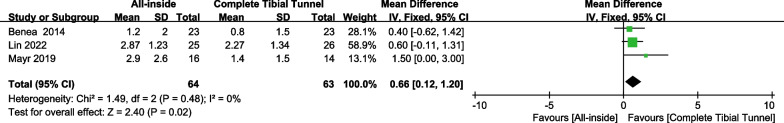
Forest plot for meta-analysis of knee laxity measured between the all-inside and complete tibial tunnel groups

Four studies [11, 20, 22, 24] investigated the anteroposterior knee stability of the operative knee using the KT-1000 arthrometer (MedMetric Corporation, San Diego, CA, USA) [11, 20, 24], and one study [22] used the GNRB arthrometer (Genourob, France). We excluded one study [20] that did not knee anterior laxity measured. All the studies stated that knee stability improved significantly postoperatively, but no significant difference between groups was noted. We found that postoperative knee stability (MD = 0.66; 95% CI, 0.12–1.20; *p* = 0.02) was comparable between the groups. (Fig. 8).

#### Graft re-rupture

**Fig. 9:**
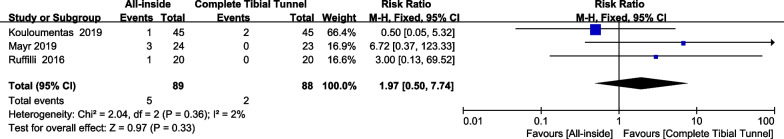
Forest plot for meta-analysis of graft re-rupture between the all-inside and complete tibial tunnel groups

Graft re-rupture was described in 3 studies [20, 24, 25].There was no significant difference within study or after pooling of data. (RR, 1.97; 95% CI,0.50–7.74; *P* = 0.33) in the All-inside group than the complete tibial tunnel group (Fig. 9).

## Discussion

The most important finding of the present study was demonstrated statistically the all-inside technique was no entirely superior to the complete tibial tunnel technique in functional outcomes, tibial tunnel widening, knee laxity measured with arthrometer, or graft re-rupture rate at final follow-up.

Volpi et al. [12] described that the clinical effects of all-inside technique and traditional reconstructive surgery were comparable for the recovery of joint motion and function outcomes Similar, but in the all-inside technique group have less risk of infection. Baldassarri et al. [19] showed that the patients who complete tibial tunnel ACLR showed slightly better performance in the postoperative 6–8 months follow-up, but this difference became insignificant in further follow-up. Connaughton et al. [9] showed that the all-inside ACL appears to have similar overall results on subjective and objective outcomes studies compared to complete tibial tunnel ACLR techniques. And the all-inside technique can decreased post-operative pain. Only 1 studies [19] reported the outcome more than 4 years. Because there were few studies with more than 2 years of follow-up, it is difficult to estimate whether the difference of treatment effect between the 2 methods will reduce over time.

The tibial tunnel healing is always a concern in ACLR surgery [27]. In the biomechanical aspect, synovial fluid penetration and micromovements at the graft to bone interface (bungee and windshield wiper effect) might enlarge the tunnel [28, 29].This defect in suspension fixation has led to the suspicion that the all-inside technique also has tunnel enlargement. All-inside technique increases mechanical wear factor, and the bone socket structure can block the accumulation of articular fluid [30]. The complete tibial tunnel technique has the destructive effect on the bone and increases the diameter of the bone, and may bring accumulation of articular fluid [30]. It is difficult to determine which causes the tunnel widening more strongly. However, one trials [24] in this meta-analysis showed that the tibial tunnel volume with button fixation was significantly smaller at all three measurement time points. The increase in the tibial tunnel volume over time was significantly larger in the group with screw fixation. Studies have confirmed that there was no significant difference in the effect with compression screws on bony canal enlargement, and the effect was even smaller than that of screws [31, 32]. Monaco et al. [31] found that tibial tunnel widening after ACLR using hamstring tendon autograft is significantly greater with complete tibial tunnel when compared to an all-inside technique at a median follow-up of 2 years. The clinical relevance of this work lies in the rebuttal of concerns arising from biomechanical studies regarding the possibility of increased tunnel widening with an all-inside technique. This was consistent with our results.

Our results showed that the knee laxity after all-inside ACLR is not better than that after tradition complete tibial tunnel ACLR, which may be related to returning to exercise prematurely after all-inside ACLR. Baldassarri et al. [19] showed that both techniques have been able to provide good clinical results. The complete tibial tunnel group, however, had a resumption of sports activity of the same level and intensity slightly longer (6.3 months) than that in the all-inside ACLR group (5.9 months). Darren et al. [10] showed that 69.2% of studies on all-inside techniques allowed cutting and rotational movements at 6–9 months postoperatively. It may be that most patients have good functional improvement at 6–9 months postoperatively. Connaughton described that a higher graft failure rate is also a concern question with the all-inside ACLR [9]. Patients return to pivoting sports prior to when the graft ligamentization process was complete as possible explanations for their high graft failure rate [33]. But this result requires longer term follow-up. In our data results showed that graft failure was no significant difference between the all-inside and complete tibial tunnel ACLR. A trials [24] showed that the all-inside technique of ACLR exhibited a trend longer operation time. The graft preparation in the above the two techniques included various preparation methods (quadrupled semitendinosus tendon, doubled gracilis and semitendinosus tendons, hamstring graft), this is an important factor that can affect clinical results. Further research is needed to determine which type of graft will yield good clinical results.

Despite a paucity of high-quality data comparing the efficacy of all-inside ACLR and complete tibial tunnel ACLR, this result suggests that all-inside ACLR may be an alternative to complete tibial tunnel ACLR. However, High-quality research is required before any conclusions can be made.

## Limitations

There are limitations to this study. First, the function of the knee joint could not be adequately assessed because of inadequate data. Second, the heterogeneity of all-inside techniques, including degree of knee flexion and external rotation of the tibial during graft types, or fixation methods across studies, may be a potential confounder of the results presented. Currently, no evidence is available in the literature that compares the outcomes of different graft types, or fixation methods. Hence, the effect of bias from this is unknown. Third, subgroup analysis according to various enhancement methods could not be performed because of insufficient literature. Fourth, only 3 trials provide complication events, limiting a entirely comparison of safety of failure between the 2 techniques. Fifth, the heterogeneity of follow-up period. Especially, a follow-up of less than 2 years is too short to evaluate the clinical outcomes of surgical treatment. Finally, the mean difference of clinical scores and tibial tunnel widening were quite small and no consider MCID (minimal clinically important difference) when interpreting the results of meta-analysis.

## Conclusion

Our meta-analysis indicated that the all-inside ACLR was superior to complete tibial tunnel ACLR in functional outcomes and tibial tunnel widening. However, the all-inside ACLR was not entirely superior to complete tibial tunnel ACLR in knee laxity measured, and graft re-rupture rate. Our systematic review and meta-analysis did not show clinically important difference in any clinical outcomes between two surgical methods and further research is required.

## Data Availability

As a meta-analysis, all raw data of this study are extracted from ten included studies. The datasets supporting the conclusions of this article are available in the 8 included studies.
